# Hydrophilicity, wettability, and elemental analysis of zirconia implant ceramic after surface conditioning methods as a function of storage time

**DOI:** 10.1007/s00784-026-06854-3

**Published:** 2026-04-06

**Authors:** Dario Philipp Luterbach, Luiza Freitas Brum Souza, Tan Fırat Eyüboğlu, Mutlu Özcan

**Affiliations:** 1 Center for Dental Medicine, Clinic of Masticatory Disorders and Dental Biomaterials, University of Zurich, Plattenstrasse 11, Zurich, CH-8032 Switzerland; 2 Faculty of Dentistry, Department of Endodontics, Istanbul Medipol University, Istanbul, Türkiye Turkey

**Keywords:** Air-abrasion, Etching, Dental materials, Laser, Oral implants, Surface conditioning, Surface free energy, Ultrasonic cleaning, Wettability, Zirconia

## Abstract

**Objectives:**

This study evaluated how distinct surface-modification protocols and storage time affect the wettability, surface free energy (SFE), and elemental composition of zirconia.

**Materials and methods:**

Zirconia specimens were subjected to six surface conditioning methods: Control (CT), alumina airborne-particle abrasion (110 μm) (AL), tribochemical silica coating (30 μm) (SI), chemical etching (ET), alumina+etching (AL-ET), and laser treatment (LS). Measurements were taken at baseline and after 1, 3, 6, and 12 months. Wettability was evaluated with water and diiodomethane contact angles to calculate work of adhesion (WoA) and surface free energy (SFE) via the Owens-Wendt-Rabel-Kaelble method. Elemental composition was assessed by energy-dispersive X-ray spectroscopy. Data analysis involved one-way ANOVA with Tukey`s post-hoc tests for treatment comparison and two-way ANOVA for storage-time effects.

**Results:**

Surface conditioning significantly affected contact angle, WoA, and SFE (p < 0.05), compared to storage. SI had the lowest water contact angles, highest WoA, and highest SFE, with a strong polar component. AL improved wettability and SFE, but less than SI. While ET showed the least hydrophilic properties at baseline but improved over time, LS was intermediate and stable. CT became more hydrophilic after 6–12 months and SI remained best. EDS confirmed these trends, with persistent silica, low aluminum after alumina treatment, and treatment-dependent elemental changes. The CT and LS spectra resembled those of the substrate.

**Conclusions:**

Both the surface-modification strategy and storage time affected zirconia wettability, with surface modification playing a greater role. While tribochemical silica coating created a durable, hydrophilic surface, Al_2_O_3_ was less effective. Etching needed time to remove residues and laser treatment offered intermediate stability.

**Clinical relevance:**

Tribochemical silica coating provided the best zirconia implant surface by maintaining a durable, hydrophilic, high surface-free energy state tha*t may promote* early protein interaction and predictable osseointegration.

## Introduction

Metal-free materials are increasingly used in reconstructive and implant dentistry. In this context, zirconia ceramics have emerged as potential substitutes for titanium implants over the last decade [1]. Its application in dentistry began in the early 1990 s, with initial studies demonstrating favorable outcomes in mechanical stability, fracture resistance, and osseointegration [2, 3]. However, implant surface properties are significantly influenced by surface conditioning and preparation methods, which play a crucial role in the success of osseointegration [4].

The clinical success of implant therapy depends heavily on the quality of the bone-implant interface, which is governed by both mechanical and biological stability. Primary stability is the mechanical interlocking achieved at implant placement in bone [5], whereas secondary stability develops over time through new bone formation and remodeling around the implant, a process known as osseointegration [6, 7]. The physicochemical properties of the implant surface, including topography, hydrophilicity, and chemical composition, play a critical role in this biological integration [4, 8].

Immediately after placement, the implant contacts blood and plasma proteins, triggering biological responses. Hydrophilicity and wettability are key surface properties influencing this process. Hydrophilicity, indicated by water contact angles below 90°, promotes blood spreading and protein adsorption, vital for clotting and healing [9]. Wettability, also measured by contact angle, reflects how well a surface can be wetted by biological fluids. Low contact angles mean high wettability, supporting protein stability and function. Hydrophobic surfaces (contact angles > 90°) may denature proteins, hindering cellular attachment and tissue integration [8].

It has been demonstrated that hydrophilic surfaces not only enhance initial protein adsorption but also promote osteoblast differentiation and maturation, thereby expediting the onset of secondary stability, which is essential for long-term implant success [4, 10]. These surface-fluid interactions are further influenced by the surface free energy (SFE), which can be divided into polar and dispersive components. Surfaces with higher polar contributions and total SFE values above 50–60 mJ/m² tend to exhibit stronger protein binding and enhanced cellular responses [11]. Typically, contact angles are measured using both polar (ultrapure water) and non-polar (diiodomethane) probe liquids to calculate SFE and work of adhesion (WoA) using the Owens-Wendt-Rabel-Kaelble (OWRK) model.

Another critical parameter is surface roughness, or microtopography, which typically spans average height values ranging from the micrometer to the sub-millimeter scale, depending on the surface-conditioning method and measurement approach [4]. Roughened surfaces increase available surface area, enhance mechanical interlocking, and thereby influence osteoblast behavior. Parameters such as Ra (average profile roughness) and Sa (average area roughness) are commonly used to quantify topography. Moderately rough surfaces with average Sa values of approximately 1–2 μm have been shown to stimulate osteoblast proliferation, differentiation, and osteogenic marker expression, thereby promoting bone formation and implant integration [4, 12–14].

Various techniques were proposed, including alumina air-abrasion, tribochemical silica coating, chemical etching, and laser structuring. The SLA (Sandblasted, Large grit, Acid-etched) protocol, commonly applied to titanium, has also been adapted from titanium, increases roughness and surface energy, aiding protein adsorption [15]. Silica coatings prepared by sol-gel methods exhibit bioactivity through calcium and phosphate ion release, promoting apatite formation and osteoconduction [16–19]. While extensively studied in titanium, evidence on zirconia’s long-term surface stability remains limited and conflicting.

Beyond immediate effects, implant surfaces may change over time during storage due to environmental exposure, hydrocarbon adsorption, and surface hydroxyl reorganization [11, 15]. These aging processes can alter wettability, surface free energy, and elemental composition, affecting early biological interactions [4, 11]. Evaluating multiple storage intervals helps assess short- and long-term stability, with extended time points revealing durability under clinical storage conditions [15].

The objective of this study was to investigate the effects of different surface modification techniques on the surface roughness, wettability, surface free energy, work of adhesion, and elemental composition of zirconia specimens across multiple storage periods. The null hypotheses tested were that (a) the surface treatment protocol would not significantly affect any of the evaluated surface characteristics compared to the untreated control group; and (b) the storage time (baseline, 1 month, 3 months, 6 months, and 1 year) would have no significant effect on any of the surface characteristics within each treatment group.

## Materials and methods

### Materials and study design

**Fig. 1:**
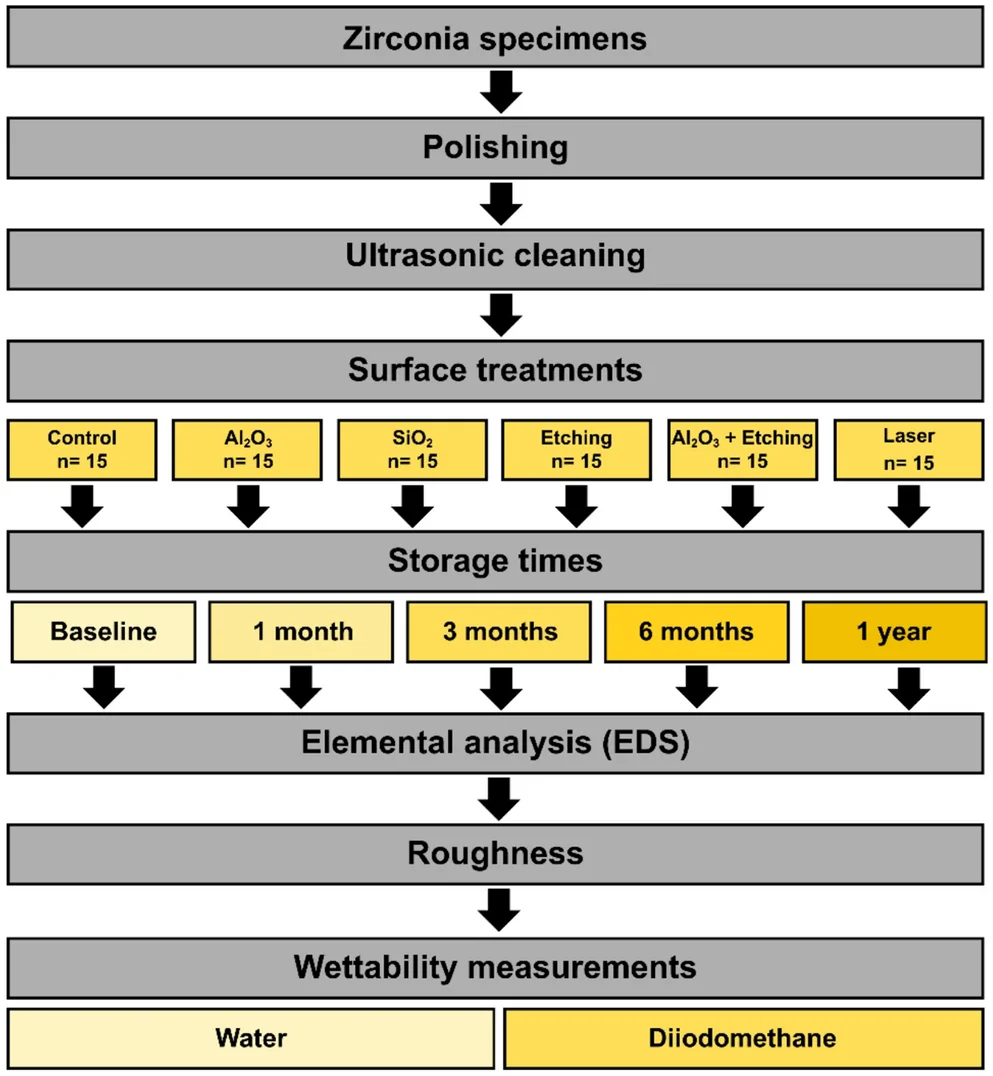
Flowchart of experimental design

The study used a factorial design with six surface conditioning protocols: Control (CT), alumina airborne-particle abrasion (AL; Al₂O₃, 110 μm), tribochemical silica coating (SI; silica-coated Al₂O₃, 30 μm), chemical etching (ET), combined alumina abrasion and etching (AL-ET), and laser treatment (LS), and five storage periods (baseline, 1 month, 3 months, 6 months, and 1 year), yielding 30 experimental groups. Each group comprised 9 zirconia specimens, for a total of 270 specimens. Details of the materials and surface modification procedures are presented in Table 1, and the complete experimental workflow, including surface characterization steps and timeline, is shown in Fig. 1.

**Table 1:** General description (brand, manufacturer, chemical composition) of materials tested

Material	Manufacturer	Composition (According to Manufacturer)
E-max ZirCAD	Ivoclar Vivadent AG, Schaan Liechtenstein	ZrO_2_ (86.0–93.5 wt%) Y_2_O_3_ (> 6.5% – ≤ 8.0 wt%) HfO_2_ (≤ 5.0 wt%) Al_2_O_3_ (≤ 1.0 wt%) and other oxides (≤ 1.0 wt%)
Rocate Pre	3 M CoJet, 3 M, St. Paul, MN, USA	Aluminium oxide (Al_2_O_3_): 98–100 wt% Sodium oxide (Na_2_O): <1 wt%
CoJet	3 M CoJet, 3 M, St. Paul, MN, USA	Aluminium oxide (> 97 wt%) Synthetic amorphous silica, fumed, crystalline free (< 5 wt%) Titanium dioxide (< 0.6 wt%)
Zircos-E	Zircos-E, RH supplies, Gaithersburg, USA	Nitric acid Hydrofluoric acid
Laser	Z-Systems AG, Oensingen, Switzerland	-

### Specimen preparation

A total of 90 zirconia specimens (Emax ZirCAD, Ivoclar, Liechtenstein; 15 × 15 × 1.5 mm^3^) were prepared for this study. All specimens were polished using a rotary polishing unit (LaboPol-21, Struers, Copenhagen, Denmark) with constant water irrigation. The polishing sequence consisted of 20 s on P#1000 followed by 20 s on P#1200 waterproof silicon carbide abrasive papers (Struers). After polishing, the specimens were cleaned in an ultrasonic bath (Telsonic TPC-25, City, Country) containing distilled water for 5 min at room temperature. After drying with compressed air, the specimens were assigned to the surface treatments.

**Fig. 2:**
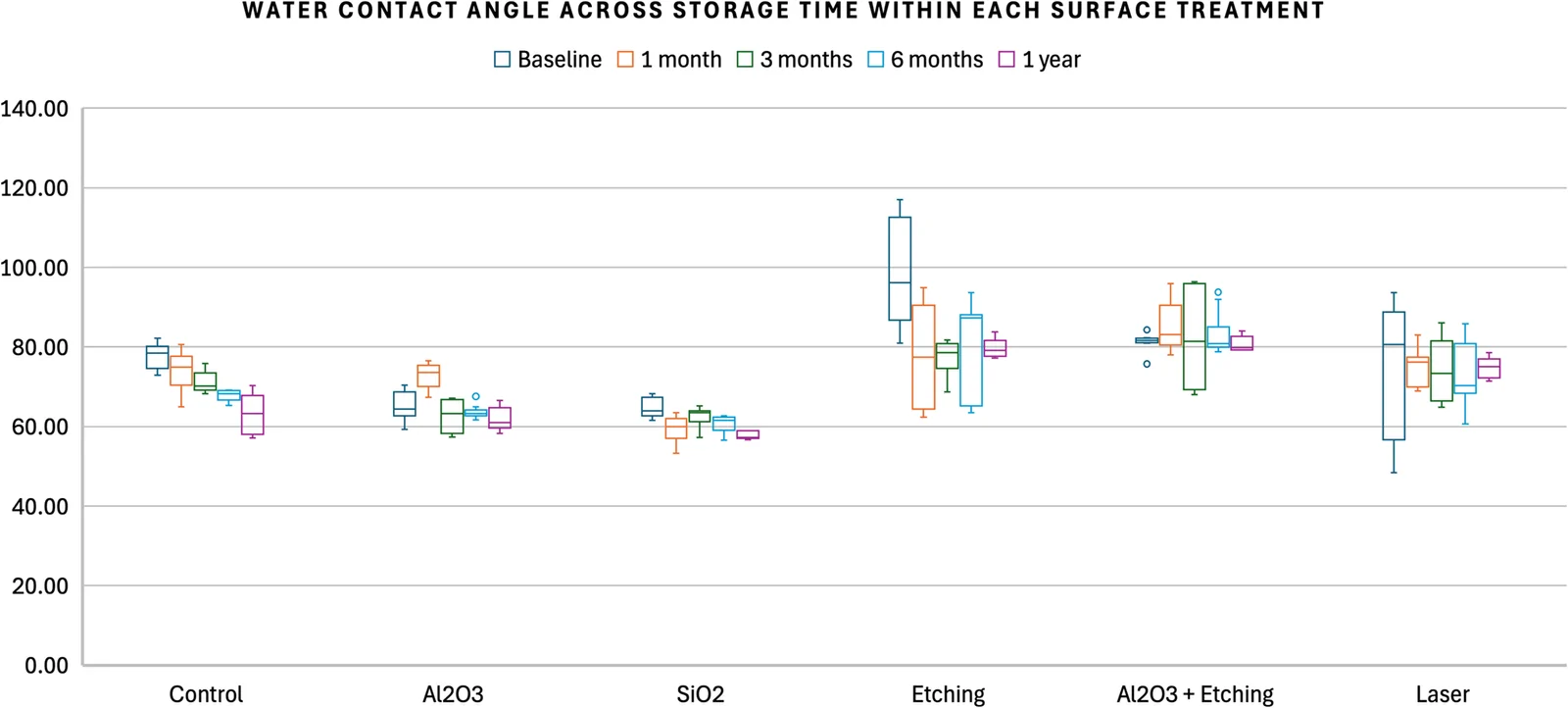
Water contact angle (θ). Grouped box plots illustrating the distribution of water contact angle values for all surface-conditioning protocols at different storage times. Within each surface treatment, storage times (Baseline, 1, 3, and 6 months, and 1 year) are displayed side by side. Boxes represent the interquartile range (IQR), horizontal black lines indicate median values, whiskers denote minimum–maximum values, and circles represent outliers. Surface treatments are color-coded for clarity

### Surface conditioning methods

The specimens were randomly divided into six groups according to the surface conditioning protocol:

- Control (CT): No additional surface modification beyond the standardized polishing and ultrasonic cleaning procedure previously described.
- Al_2_O_3_ (AL): The surface was roughened with Al_2_O_3_ (3 M Rocatec Pre, 3 M ESPE, Minn, USA, 110 μm particles) at 2.5 bar from a distance of 10 mm for 20 s. After blasting, the specimens were ultrasonically cleaned in distilled water for 5 min and then air-dried.
- SiO_2_ (SI): Surface modification was performed with silica-coated alumina (3 M CoJet system, 30 μm particles) at 2.5 bar from a distance of 1 cm for 20 s. These specimens underwent the same cleaning and drying procedure as the Al_2_O_3_ group.
- Etching (ET): Specimens were conditioned with Zircos-E etching solution (RH Supplies, Gaithersburg, MD, USA) by immersion and placed in an ultrasonic bath at frequencies between 20 and 60 kHz and a power density of 0.2 to 1 W/cm² at room temperature for 30 min. After rinsing under running water for 3 min, the specimens were steam-dried and subjected to a degassing cycle in a porcelain furnace at 800 °C for 1 min using a glaze program.
- Al_2_O_3_ + etching (AL-ET): The specimens underwent a combined protocol, first air-abraded with alumina particles as described for the Al_2_O_3_ group and then treated with the etching protocol described above.
- 6Laser (LS): The specimens were subjected to surface modification by laser irradiation (Z-Systems AG, Oensingen, Switzerland), a company specializing in laser-treated zirconia implant surfaces. The specific laser parameters and processing details were proprietary and not disclosed.

### Storage

Specimens from control and treated groups were randomly assigned to five storage periods before analysis: baseline, 1 month, 3 months, 6 months, and 1 year. Baseline specimens were analyzed immediately after surface conditioning, whereas the remaining specimens were stored at room temperature under dry conditions for their designated time intervals.

### Energy-dispersive X-ray spectroscopy

After each surface treatment and storage period, the specimens were analyzed using a tabletop scanning electron microscope (TM4000Plus, Hitachi, Tokyo, Japan). Surface morphology was assessed in secondary electron (SE) mode at an accelerating voltage of 15 kV and a working distance of approximately 10 mm. Elemental chemical composition was evaluated by energy-dispersive X-ray spectroscopy (EDS) using the integrated EDS system with a silicon drift detector (SDD). EDS analyses were performed in mapping mode at 15 kV to qualitatively assess the spatial distribution of elements on the specimen surfaces.

### Wettability and surface free energy

Surface wettability was evaluated by measuring the static contact angle (θ) with a contact angle goniometer (Krüss Easy Drop, Krüss GmbH, Hamburg, Germany) and Drop Shape Analysis software. Each specimen was first analyzed with distilled water and then with 99% diiodomethane, both dispensed as 1 µL droplets. These test liquids were selected from the internal Liquid Database of the software, which automatically applies the corresponding physical parameters.

For each liquid, the software calculated the contact angle (θ) and the work of adhesion (WoA) using the following equation:

$$\:\mathrm{W}\mathrm{o}\mathrm{A}=\gamma_L(1+\mathrm{cos}\theta\:)$$

where $\:\gamma_L$ is the surface tension of the liquid, and $\:\theta\:$ is the contact angle between the droplet and the solid surface.

By performing the measurements with two liquids of known surface tension components (one polar, one non-polar), a system of two equations is obtained and solved to determine the surface energy components for each specimen. The surface free energy of the solid ($\:\gamma_S$) and its components were calculated using the Owens-Wendt-Rabel-Kaelble (OWRK) model, which separates the total surface energy into a dispersive component ($\:{\gamma\:}_S^d$) and a polar component ($\:{\gamma\:}_S^p$). The model assumes that:

$$\:\gamma_S={\gamma\:}_S^d+{\gamma\:}_S^p$$

and is based on the equation:

$$\:\gamma_L\left(1+\mathrm{cos}\theta\:\right)=2\left(\sqrt{{\gamma\:}_S^d{\gamma\:}_L^d}+\sqrt{{\gamma\:}_S^p{\gamma\:}_L^p}\right)$$

where: $\:{\gamma\:}_L^d$ and $\:{\gamma\:}_L^p$ are the dispersive and polar components of the surface tension of the liquid, and $\:{\gamma\:}_S^d$ and $\:{\gamma\:}_S^p$ are the unknown dispersive and polar components of the surface energy or the solid. The test liquids used and their surface tension properties were: Distilled water: $\:\gamma_L$= 72.8mN/m, $\:{\gamma\:}_{L}^{d}$= 26.4 mN/m, and $\:{\gamma\:}_{L}^{p}$= 46.4 mN/m; Diiodomethane (99%): $\:\gamma_L$= 50.8 mN/m, with $\:{\gamma\:}_{L}^{d}$= 50.8 mN/m and $\:{\gamma\:}_{L}^{p}$= 0 mN/m.

All measurements were conducted under controlled ambient conditions. The results were reported as the mean of nine measurements per group, and the total surface energy $\left(\gamma_S\right)$, as well as its polar and dispersive components, were reported in mN/m.

### Surface roughness analysis

A micrometric roughness analysis was performed (*n* = 3) using a 3D digital microscope (VHX-7000, Keyence Co., Osaka, Japan) *equipped with a 300× magnification objective*. Three-dimensional surface images were acquired, and profile lines were traced on the reconstructed 3D topographies to determine the differences between the smallest and largest surface undulations, which were expressed as roughness values. For each specimen, three measurements were performed along each axis (x and y), using a cut-off length (λc) of 0.25 mm and a sampling length of 1.25 mm. The arithmetic mean of the measurements was calculated for each specimen in accordance with ISO 21920-2 (2021), considering the Ra (arithmetical mean surface roughness) parameter.

### Statistical analysis

Since the data for surface roughness, contact angle, work of adhesion, and surface free energy met normality and homogeneity of variance assumptions, verified by the Shapiro-Wilk and Levene’s tests (*p* > 0.05), parametric analyses were used (α = 0.05) with SPSS (version 21, IBM). Surface roughness effects from conditioning and storage time, and their interaction, were analyzed using two-way ANOVA, with Tukey’s post hoc test for significant effects. Contact angle, work of adhesion, and surface free energy were assessed using one-way ANOVA because surface free energy and work of adhesion are derived parameters calculated from paired contact angle measurements according to the Owens-Wendt-Rabel-Kaelble method. Contact angle, work of adhesion, and surface free energy were assessed with one-way ANOVA at each storage time and surface conditioning, plus pairwise comparisons with Tukey’s test. This approach was applied to evaluate treatment-specific and storage-time-dependent changes in wettability. Differences among storage times within each group were also analyzed via one-way ANOVA and Tukey’s post hoc. Elemental data were reported descriptively. Effect sizes, as eta squared (η²), were calculated for all ANOVA models. Effect sizes were interpreted as small (η² ≈ 0.01), moderate (η² ≈ 0.06), or large (η² ≥ 0.14). Elemental composition data were analyzed descriptively and qualitatively.

## Results

**Fig. 3:**
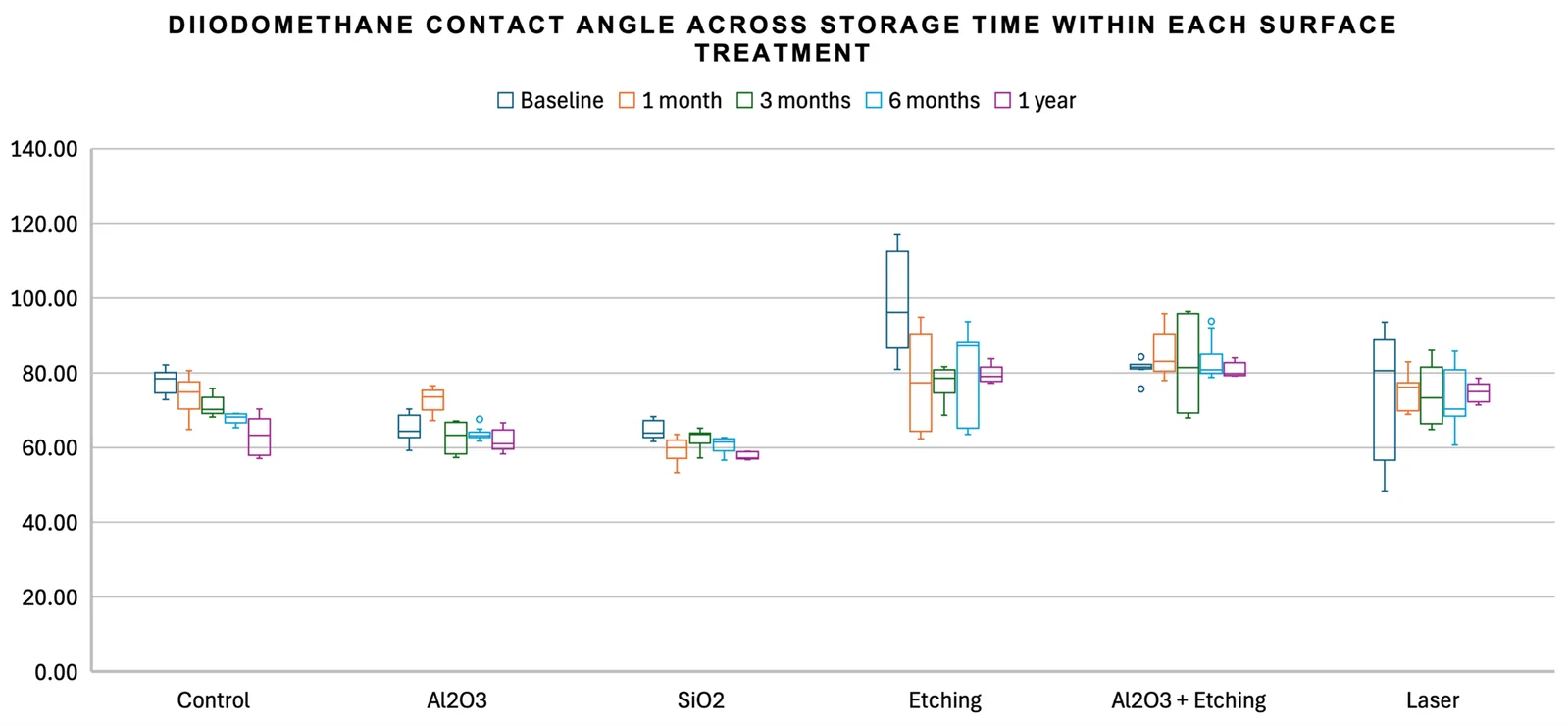
Diiodomethane contact angle. Grouped box plots showing diiodomethane contact angle values as a function of surface-conditioning protocol and storage time. Storage times are presented side by side within each surface treatment group. Boxes represent the interquartile range, horizontal black lines indicate medians, whiskers denote minimum–maximum values, and circles indicate outliers. Colors correspond to different surface treatments

**Fig. 4:**
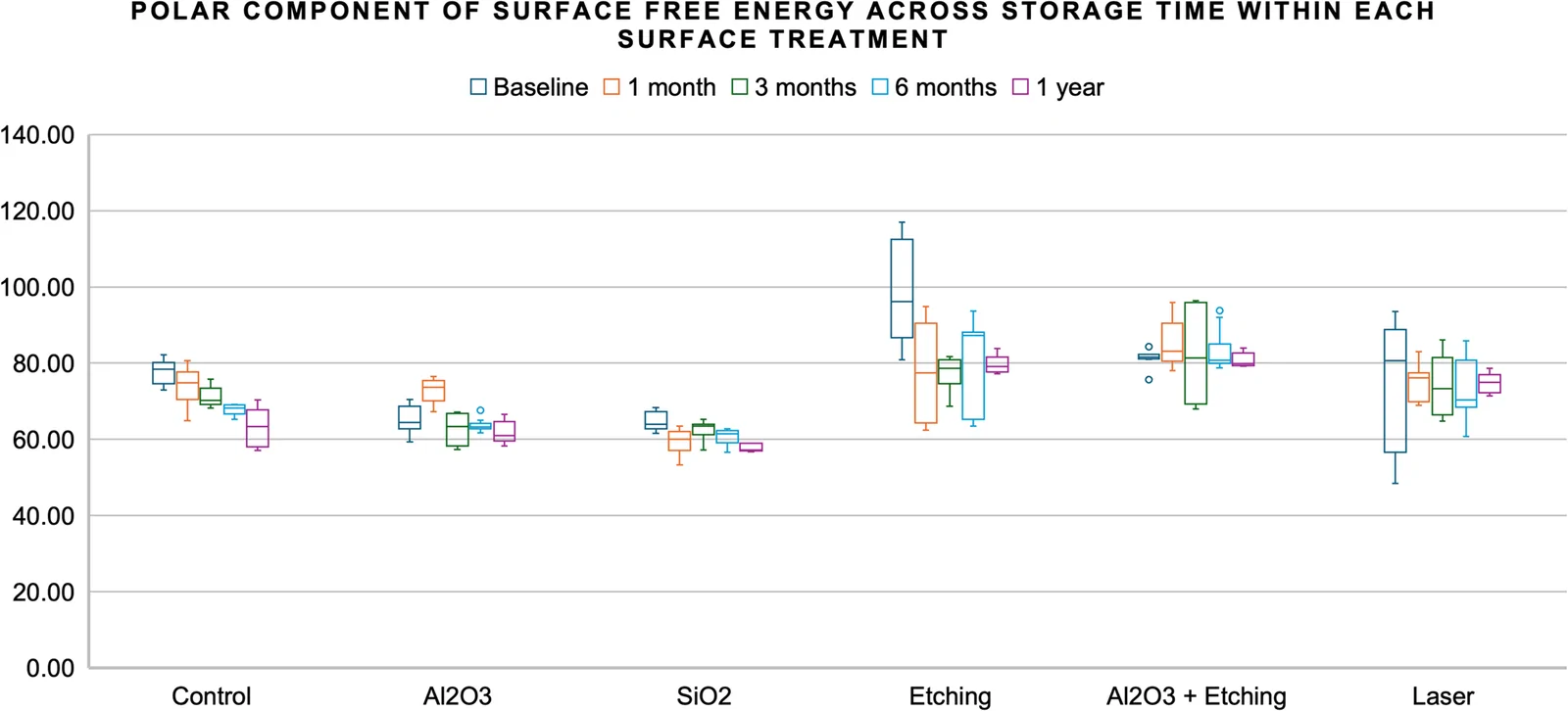
Polar component of surface free energy (γSp). Grouped box plots of the polar component of surface free energy (γSp) for all surface-conditioning protocols across different storage times. Storage periods are shown side by side within each surface treatment. Boxes represent the interquartile range, black horizontal lines indicate median values, whiskers denote minimum–maximum values, and circles represent outliers. Different colors denote different surface treatments

**Fig. 5:**
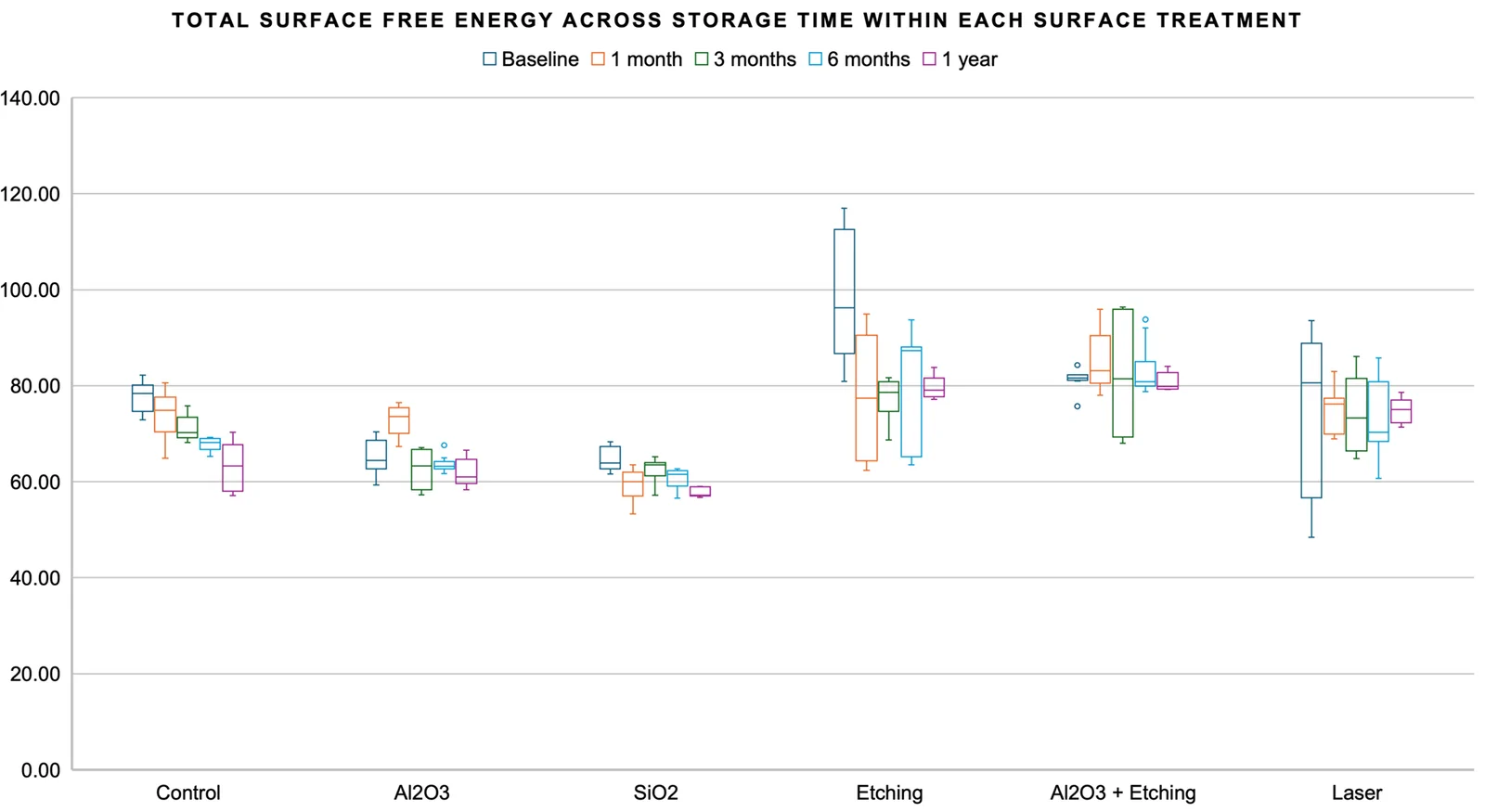
Total surface free energy (γS). Grouped box plots depicting total surface free energy (γS) values for the investigated surface-conditioning protocols at various storage times. Storage times are displayed side by side within each surface treatment group. Boxes represent the interquartile range, horizontal black lines indicate median values, whiskers denote minimum–maximum values, and circles indicate outliers. Surface treatments are distinguished by color coding

For water contact angle (θ), surface conditioning significantly influenced the results at all storage times with a large effect size (*p* < 0.05, η² = 0.41) (Table 2). *SI c*onsistently exhibited the lowest θ values, whereas *ET and AL-ET* showed the highest values. *CT and LC gro*ups demonstrated intermediate values. *AL gen*erally showed lower θ values *than CT and ET* and was statistically *similar to SI at* all storage times except at 1 month. The corresponding water-derived work of adhesion (WoA) followed the same ranking pattern among surface-conditioning protocols (*p* < 0.05, η² = 0.39). *T*o facilitate simultaneous visualization of treatment-related effects and storage-time–dependent trends, grouped box plots were generated for water contact angle and surface free energy parameters, with all storage times displayed side by side within each surface-conditioning protocol (Figs. 2, 3, 4 and 5)*.*

**Fig. 6:**
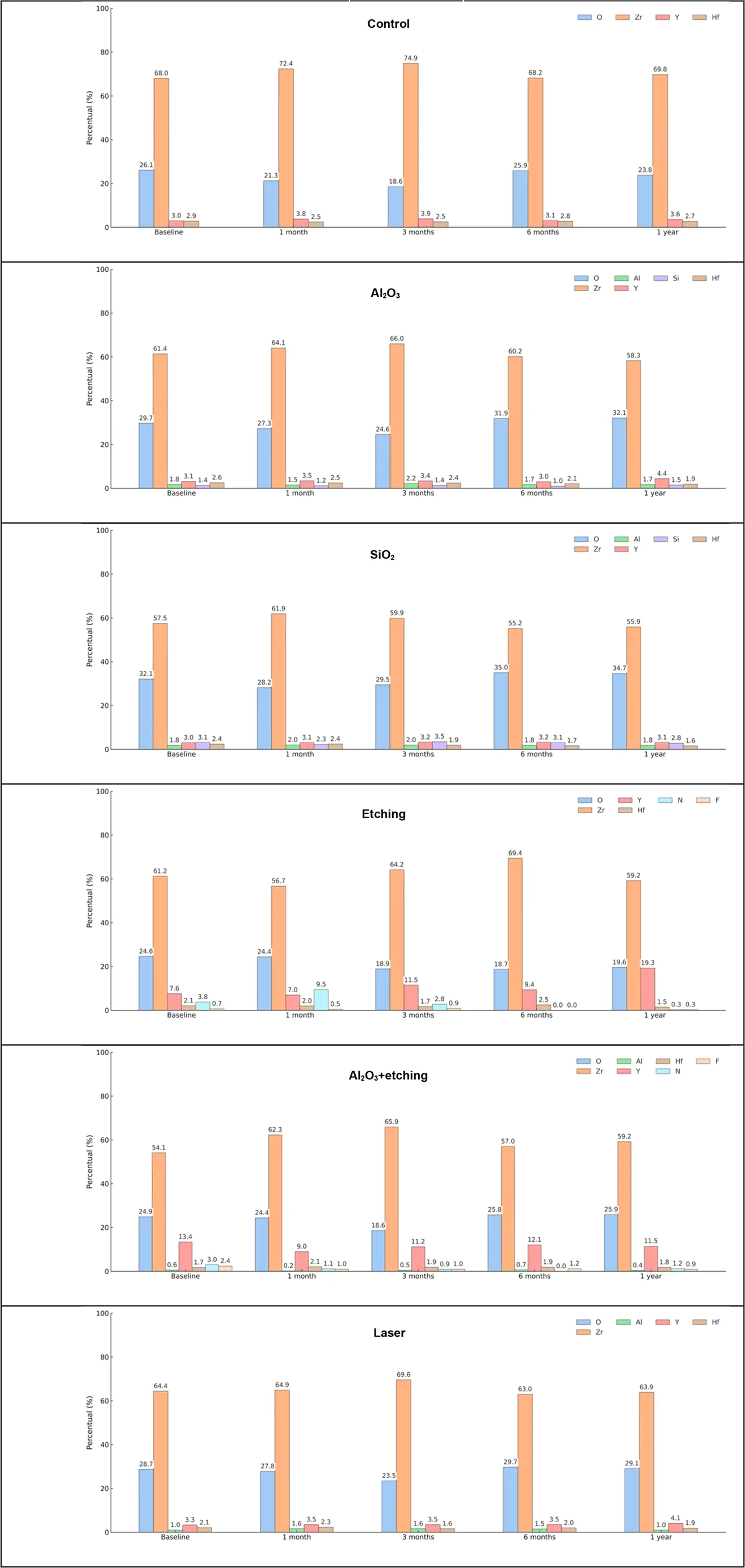
Elemental analysis results by surface treatment

**Table 2:** Means and standard deviations for Contact angle (degrees) and Work of Adhesion (mN/m)

Storage time	Surface treatment	Water		Diiodomethane	
		Contact Angle (θ) Mean ± SD	Work of Adhesion (WoA) Mean ± SD	Contact Angle (θ) Mean ± SD	Work of Adhesion (WoA) Mean ± SD
Baseline	Control	77.64 ±3.17 ^BC^	88.36 ±3.93 ^BC^	50.79 ±2.16 ^AB^	82.89 ±1.48 ^BC^
	Al_2_O_3_	65.22 ±3.57 ^CD^	103.26 ±4.11 ^AB^	47.04 ±5.92 ^B^	85.25 ±3.77 ^B^
	SiO_2_	64.77 ±2.42 ^D^	103.81 ±2.79 ^A^	50.17 ±2.41 ^B^	83.31 ±1.63 ^B^
	Etching	98.40 ±13.00 ^A^	62.43 ±16.07 ^D^	55.43 ±1.47 ^A^	79.62 ±1.08 ^C^
	Al_2_O_3_ + Etching	81.29 ±4.96 ^B^	83.82 ±2.89 ^C^	40.09 ±4.29 ^C^	89.57 ±2.46 ^A^
	Laser	74.73 ± 16.80 ^BCD^	91.12 ±19.78 ^ABC^	46.47 ±4.08 ^B^	85.71 ±2.68 ^B^
1 month	Control	73.94 ±4.97 ^B^	92.86 ±6.02 ^B^	51.14 ±1.99 ^A^	82.65 ±1.36 ^B^
	Al_2_O_3_	72.74 ±3.22 ^B^	94.36 ±3.89 ^B^	51.12 ±2.91 ^A^	82.65 ±2.03 ^B^
	SiO_2_	59.47 ±3.33 ^C^	109.73 ±3.60 ^A^	50.90 ±0.46 ^A^	82.84 ±0.32 ^B^
	Etching	77.81 ±12.35 ^AB^	87.86 ±15.20 ^BC^	50.62 ±5.23 ^A^	82.91 ±3.66 ^B^
	Al_2_O_3_ + Etching	84.84 ±6.44 ^A^	79.32 ±8.15 ^C^	48.88 ±8.10 ^A^	83.91 ±5.21 ^B^
	Laser	74.82 ±4.64 ^B^	91.81 ±5.68 ^B^	42.02 ±3.13 ^B^	88.49 ±1.84 ^A^
3 months	Control	71.11 ±2.79 ^BC^	96.34 ±3.38 ^AB^	51.19 ±1.54 ^A^	82.63 ±1.06 ^B^
	Al_2_O_3_	62.48 ±3.99 ^C^	106.37 ±4.48 ^A^	48.46 ±2.73 ^A^	84.45 ±1.82 ^B^
	SiO_2_	62.52 ±2.38 ^C^	106.36 ±2.65 ^A^	49.52 ±2.76 ^A^	83.74 ±1.85 ^B^
	Etching	77.31 ±4.27 ^AB^	88.75 ±5.24 ^BC^	43.04 ±4.03 ^B^	87.84 ±2.49 ^A^
	Al_2_O_3_ + Etching	81.56 ±12.02 ^A^	83.30 ±15.00 ^C^	43.93 ±3.27 ^B^	87.33 ±2.03 ^A^
	Laser	74.06 ±7.73 ^AB^	92.64 ±9.44 ^BC^	43.27 ±2.65 ^B^	87.76 ±1.65 ^A^
6 months	Control	67.77 ±1.42 ^CD^	100.34 ±1.67 ^AB^	51.19 ±1.84 ^A^	82.62 ±1.26 ^B^
	Al_2_O_3_	63.70 ±1.81 ^CD^	105.04 ±2.08 ^AB^	53.12 ±3.94 ^A^	81.22 ±2.73 ^B^
	SiO_2_	60.70 ±2.11 ^D^	108.40 ±2.31 ^A^	52.33 ±2.80 ^A^	81.81 ±1.97 ^B^
	Etching	80.38 ±12.22 ^AB^	84.67 ±15.00 ^CD^	49.04 ±2.89 ^A^	84.06 ±1.92 ^AB^
	Al_2_O_3_ + Etching	83.53 ±5.60 ^A^	80.98 ±7.09 ^D^	48.92 ±4.24 ^AB^	84.10 ±2.86 ^AB^
	Laser	72.76 ±8.41 ^BC^	94.18 ±10.19 ^BC^	44.50 ±3.10 ^B^	86.99 ±1.94 ^A^
1 year	Control	63.47 ±4.96 ^C^	105.21 ±5.62 ^B^	51.07 ±2.77 ^AB^	82.69 ±1.93 ^AB^
	Al_2_O_3_	61.96 ±2.87 ^CD^	106.99 ±3.24 ^AB^	49.41 ±2.38 ^B^	83.83 ±1.61 ^A^
	SiO_2_	59.09 ±1.69 ^D^	111.27 ±1.85 ^A^	47.54 ±1.65 ^B^	85.08 ±1.08 ^A^
	Etching	79.60 ±2.27 ^A^	85.93 ±2.84 ^D^	47.74 ±3.51 ^B^	84.90 ±2.33 ^A^
	Al_2_O_3_ + Etching	80.82 ±1.95 ^A^	84.41 ±2.45 ^D^	54.59 ±1.94 ^A^	80.22 ±1.40 ^B^
	Laser	74.86 ±2.64 ^B^	91.80 ±3.23 ^C^	47.54 ±3.24 ^B^	85.04 ±2.16 ^A^

*Uppercase letters indicate statistical differences between different surface conditioning and storage times (1-way ANOVA and Tukey’s HSD post-hoc tests (α = 0.05)

For diiodomethane contact angle, surface conditioning also had a significant effect at all storage times with a moderate-to-large effect size (*p* < 0.05, η² = 0.24). At baseline, *SI and AL* showed the lowest θ values and were not statistically different. At 1 month, *SI* exhibited significantly lower θ values than *AL.* At 3 and 6 months, *SI and A*L became statistically similar and showed lower θ values than *ET and AL-ET*. At 12 months, *SI* again demonstrated significantly lower θ values *than AL.* The corresponding diiodomethane-derived WoA values showed consistent trends (*p* < 0.05, η² = 0.22).

Total surface free energy (σ) was significantly affected by surface conditioning at all storage times with a large effect size (*p* < 0.05, η² = 0.46) (Table 3). *SI* consistently showed the highest σ values across all time points. *AL* presented σ values comparable to *SI* at Baseline, 3, 6, and 12 months but not at 1 month. *CT* specimens demonstrated a progressive increase in σ over time. *ET and AL-ET* exhibited the lowest σ values, while *LS* showed intermediate and temporally stable values.

**Table 3:** Means and standard deviations of surface energy parameters in mN/m

Storage time	Surface treatment	σ^d^ (Dispersive Component)	σ^*p*^ (Polar Component)	σ (Total Surface Energy)
Baseline	Control	33.82 ±1.21 ^BC^	13.47 ±3.25 ^BC^	47.30 ±3.11 ^B^
	Al_2_O_3_	35.83 ±3.18 ^B^	26.04 ±5.56 ^A^	61.87 ±4.45 ^A^
	SiO_2_	34.17 ±1.34 ^B^	27.86 ±2.84 ^A^	62.03 ±3.16 ^A^
	Etching	31.20 ±0.84 ^C^	3.80 ±3.76 ^C^	35.00 ±4.18 ^C^
	Al_2_O_3_ + Etching	39.51 ±2.16 ^A^	7.38 ±2.22 ^BC^	46.89 ±2.03 ^B^
	Laser	36.18 ±2.24 ^B^	18.67 ±19.72 ^AB^	54.85 ±17.68 ^AB^
1 month	Control	33.63 ±1.11 ^B^	17.58 ±5.72 ^B^	51.21 ±5.54 ^BC^
	Al_2_O_3_	33.64 ±2.64 ^B^	18.79 ±4.63 ^B^	52.42 ±3.50 ^B^
	SiO_2_	33.77 ±0.26 ^B^	35.40 ±4.54 ^A^	69.17 ±4.66 ^A^
	Etching	33.89 ±2.94 ^B^	14.69 ±10.47 ^BC^	48.58 ±12.73 ^BC^
	Al_2_O_3_ + Etching	34.76 ±4.36 ^B^	7.25 ±3.52 ^C^	42.02 ±5.96 ^C^
	Laser	38.55 ±1.60 ^A^	13.41 ±3.91 ^BC^	51.96 ±4.69 ^B^
3 months	Control	33.61 ±0.87 ^B^	20.56 ±3.34 ^B^	54.17 ±3.15 ^B^
	Al_2_O_3_	35.12 ±1.51 ^B^	30.07 ±5.13 ^A^	65.19 ±5.31 ^A^
	SiO_2_	34.53 ± 1.53 ^B^	30.47 ±2.54 ^A^	64.99 ±3.24 ^A^
	Etching	38.00 ± 2.13 ^A^	11.48 ±4.07 ^C^	49.48 ±4.03 ^B^
	Al_2_O_3_ + Etching	37.55 ± 1.74 ^A^	10.38 ±9.07 ^C^	47.94 ±7.95 ^B^
	Laser	37.91 ± 1.41 ^A^	14.94 ±6.86 ^BC^	52.85 ±7.85 ^B^
6 months	Control	33.60 ±1.03 ^B^	24.53 ±2.22 ^BC^	59.14 ±1.74 ^BC^
	Al_2_O_3_	32.50 ±2.21 ^B^	30.90 ±3.40 ^AB^	63.40 ±2.34 ^AB^
	SiO_2_	32.95 ±1.58 ^B^	34.51 ±2.93 ^A^	67.47 ±2.92 ^A^
	Etching	34.79 ±1.60 ^AB^	12.34 ±11.66 ^D^	47.13 ±11.69 ^DE^
	Al_2_O_3_ + Etching	34.84 ±2.35 ^AB^	8.40 ±4.26 ^D^	43.24 ±2.82 ^E^
	Laser	37.25 ±1.65 ^A^	17.07 ±8.81 ^CD^	54.32 ±8.09 ^CD^
1 year	Control	33.67 ±1.56 ^AB^	30.32 ±7.89 ^A^	63.99 ±6.63 ^B^
	Al_2_O_3_	34.59 ±1.32 ^A^	31.24 ±4.33 ^A^	65.83 ±3.80 ^B^
	SiO_2_	35.63 ±0.91 ^A^	35.42 ±2.85 ^A^	71.05 ±2.27 ^A^
	Etching	35.50 ±1.93 ^A^	10.68 ±2.56 ^B^	46.18 ±1.68 ^CD^
	Al_2_O_3_ + Etching	31.68 ±1.11 ^B^	11.71 ±1.91 ^B^	43.39 ±1.79 ^D^
	Laser	35.61 ±1.79 ^A^	15.10 ±3.36 ^B^	50.71 ±2.52 ^C^

*Uppercase letters indicate statistical differences between different surface treatments and storage times (1-way ANOVA and Tukey`s HSD post-hoc tests (α = 0.05)

The polar component of surface free energy (σᵖ) was significantly influenced by surface conditioning with a large effect size (*p* < 0.05, η² = 0.49). *SI* showed the highest σᵖ values at all storage times, whereas *ET and AL-ET* exhibited consistently low σᵖ values. The dispersive component (σᵈ) was also significantly affected by surface conditioning, with a moderate effect size (*p* < 0.05, η² = 0.18), indicating smaller between-group variability than for σᵖ. Effect size analysis indicated that the surface-conditioning protocol accounted for a substantially larger proportion of variance in surface free energy parameters than storage time.

Storage time significantly affected water contact angle and total surface energy with moderate effect sizes (θ: *p* < 0.05, η² = 0.09; σ: *p* < 0.05, η² = 0.12) (Tables 4, 5, 6, 7, 8 and 9). In the *CT* group, θ values decreased significantly at 6 months and 1 year compared with Baseline. In the *AL* group, θ was significantly higher at 1 month than at all other storage times. In the *SI* group, Baseline θ values were significantly higher than those at 1 month and 1 year, and 3-month values were significantly higher than at 1 year. In the *ET* group, Baseline θ values were significantly higher than those at all subsequent storage times. In the *AL-ET* group, θ was significantly higher at 1 month than at Baseline and higher at 3 months than at 1 month. No significant differences in θ across storage times were observed for the *LS* group (*p* > 0.05, η² = 0.01).

**Table 4:**
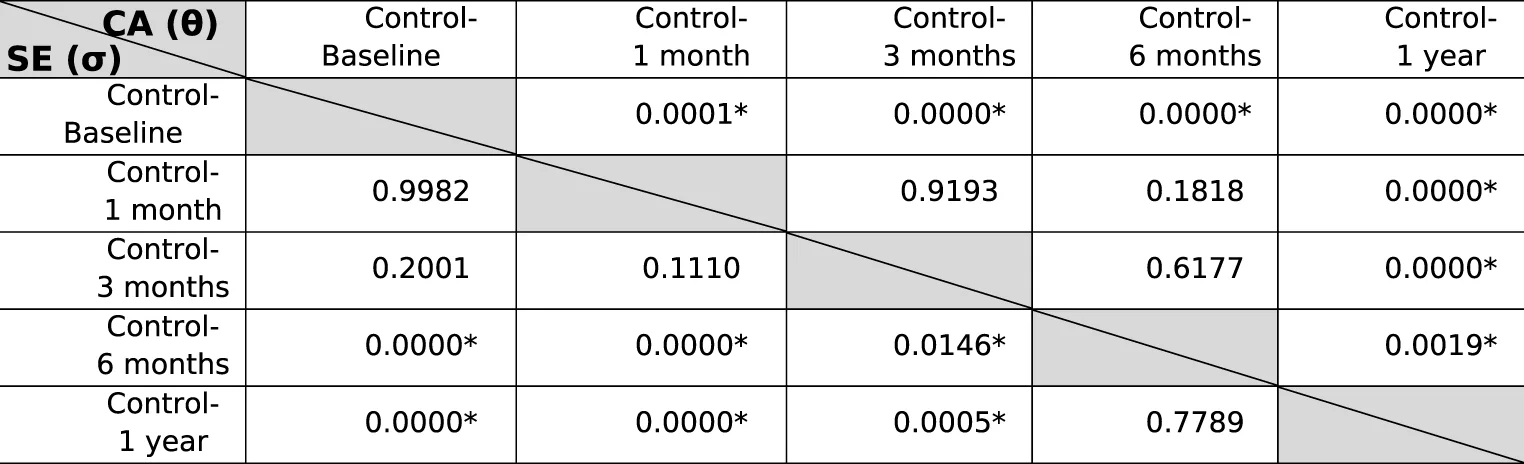
*P* values from Tukey’s post-hoc test indicating differences in water Contact Angle (θ) and Total Surface Energy (σ) between storage times for the Control group

**Table 5:**
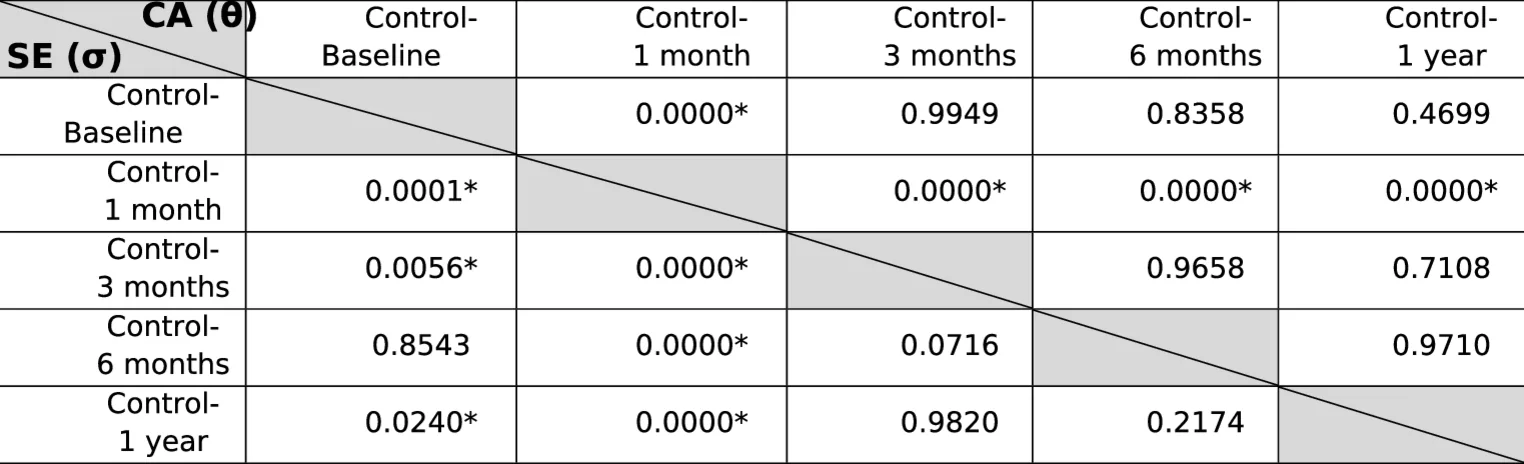
*P* values from Tukey’s post-hoc test indicating differences in water Contact Angle (θ) and Total Surface Energy (σ) between storage times for the Al_2_O_3_ group

**Table 6:**
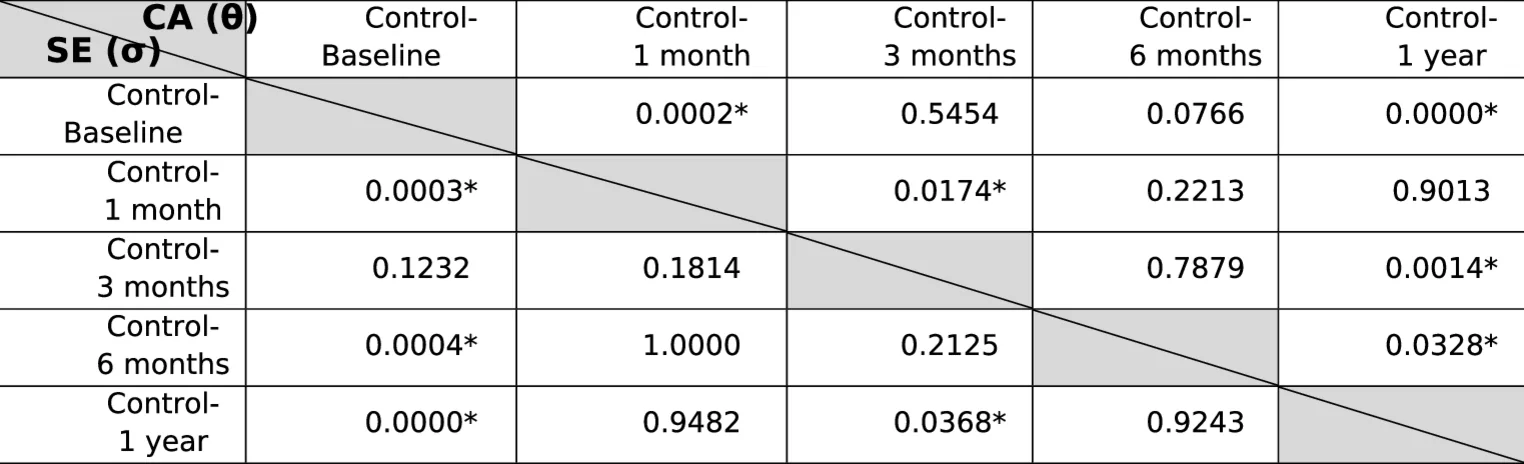
*P* values from Tukey’s post-hoc test indicating differences in water Contact Angle (θ) and Total Surface Energy (σ) between storage times for the SiO_2_ group

**Table 7:**
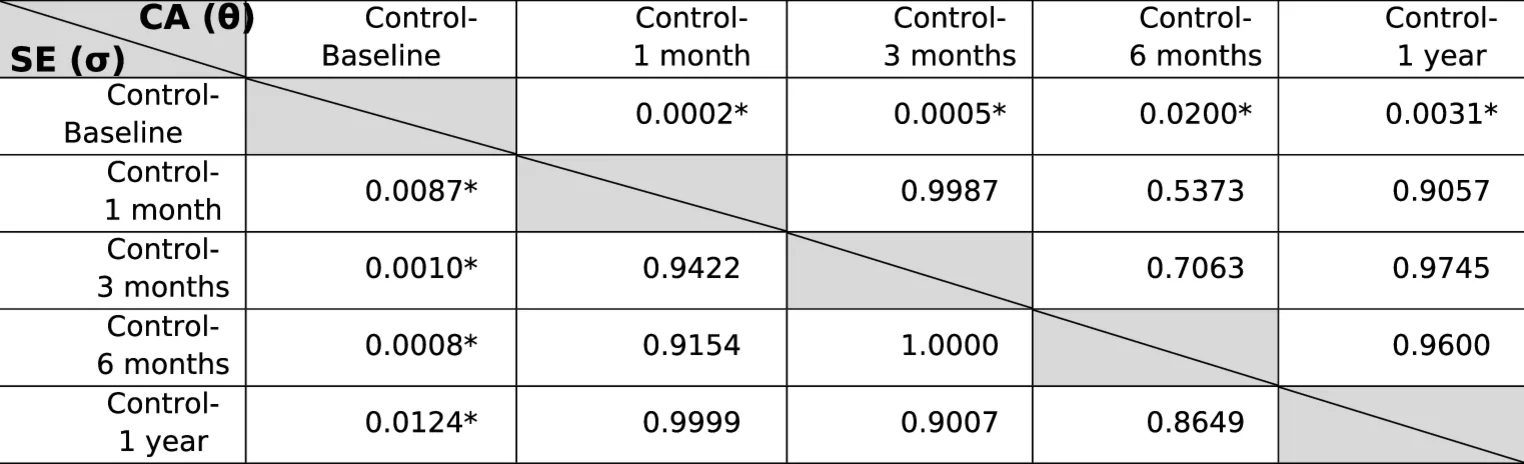
*P* values from Tukey’s post-hoc test indicating differences in water Contact Angle (θ) and Total Surface Energy (σ) between storage times for the Etching group

**Table 8:**
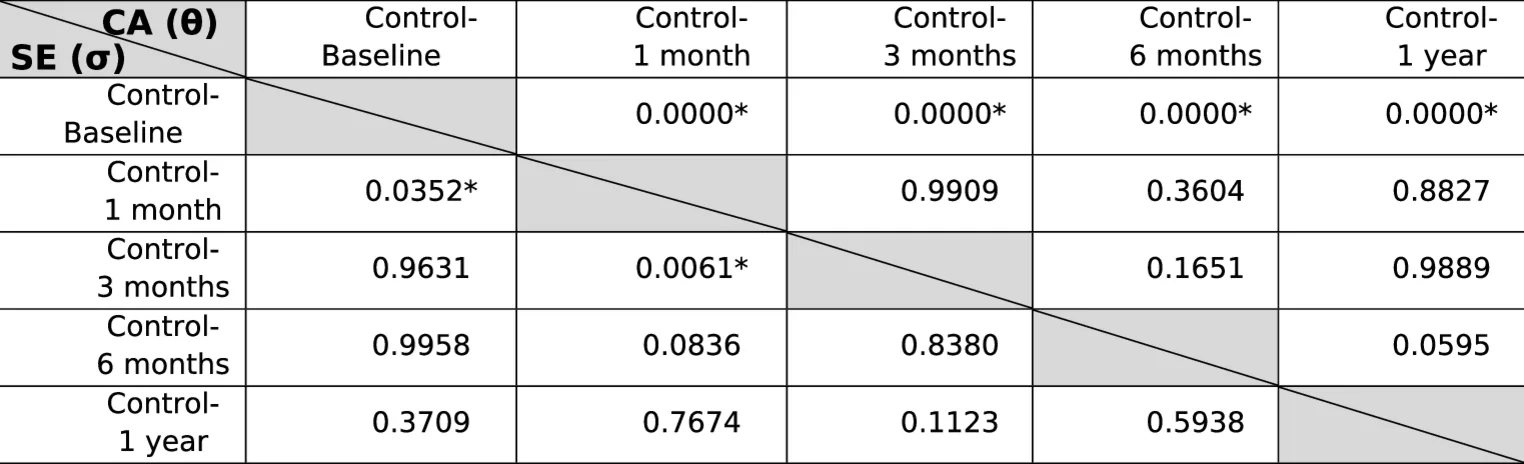
*P* values from Tukey’s post-hoc test indicating differences in water Contact Angle (θ) and Total Surface Energy (σ) between storage times for the Al_2_O_3_+Etching group

**Table 9:**
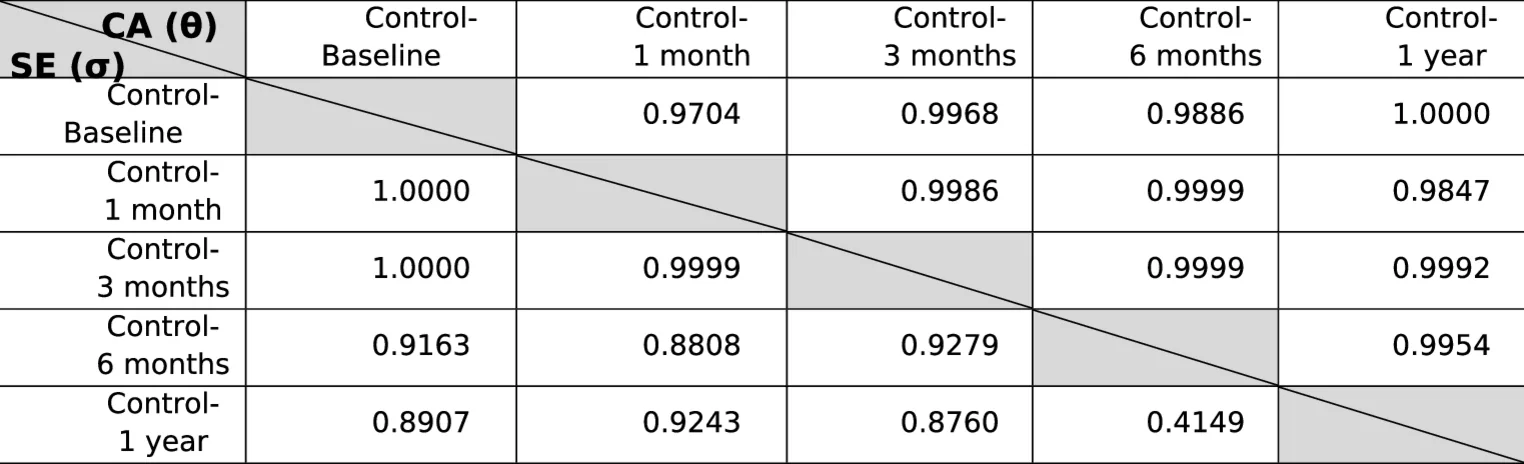
*P* values from Tukey’s post-hoc test indicating differences in water Contact Angle (θ) and Total Surface Energy (σ) between storage times for the Laser group

For total surface energy (σ), *CT* specimens showed significantly higher values at 6 months and 1 year compared with Baseline. In the *AL* group, Baseline σ values were significantly higher than those at 1 month, with no differences among 3, 6, and 12 months. In the *SI* group, σ at 1 month was significantly higher than at Baseline, while values at 3 and 6 months were significantly higher than at 1 year. In the *ET* group, Baseline σ values were significantly lower than those at all later storage times. In the *AL-ET*, Baseline σ values were significantly higher than those at all other storage times. No significant storage-time effect on σ was detected for the LS group (*p* > 0.05, η² = 0.01).

Surface roughness (Ra) was significantly influenced by surface-conditioning protocol with a very large effect size (*p* < 0.001, η² = 0.93) (Table 10). Laser-treated specimens exhibited significantly higher Ra values than all other surface treatments. No significant differences in Ra were observed among *CT*,* AL*,* SI*,* ET and AL-ET* groups. Storage time had no significant effect on surface roughness (*p* > 0.05, η² = 0.01), and no significant interaction between surface conditioning and storage time was detected (η² = 0.01).

**Table 10:** Surface roughness (Ra, µm) values for all surface-conditioning protocols and storage times, expressed as mean ± standard deviation (SD). Different uppercase letters (A, B) indicate statistically significant differences among surface-conditioning protocols within the same storage time (two-way ANOVA followed by Tukey’s HSD post hoc test, α = 0.05). No statistically significant differences in surface roughness were observed among storage times within any surface-conditioning protocol

Surface treatment	Baseline	1 month	3 months	6 months	1 year
**Control**	0.26 ± 0.04 ^**B**^	0.25 ± 0.05 ^**B**^	0.26 ± 0.04 ^**B**^	0.25 ± 0.05 ^**B**^	0.26 ± 0.04 ^**B**^
**Al₂O₃**	0.12 ± 0.02 ^**B**^	0.13 ± 0.02 ^**B**^	0.12 ± 0.02 ^**B**^	0.12 ± 0.03 ^**B**^	0.13 ± 0.02 ^**B**^
**SiO₂**	0.17 ± 0.04 ^**B**^	0.17 ± 0.03 ^**B**^	0.18 ± 0.04 ^**B**^	0.17 ± 0.03 ^**B**^	0.17 ± 0.04 ^**B**^
**Etching**	0.15 ± 0.04 ^**B**^	0.14 ± 0.04 ^**B**^	0.15 ± 0.03 ^**B**^	0.15 ± 0.04 ^**B**^	0.14 ± 0.03 ^**B**^
**Al₂O₃ + Etching**	0.14 ± 0.04 ^**B**^	0.14 ± 0.03 ^**B**^	0.15 ± 0.04 ^**B**^	0.14 ± 0.03 ^**B**^	0.14 ± 0.03 ^**B**^
**Laser**	3.02 ± 0.26 ^**A**^	3.01 ± 0.27 ^**A**^	3.03 ± 0.25 ^**A**^	3.00 ± 0.28 ^**A**^	3.02 ± 0.26 ^**A**^

Elemental analysis (EDS) (Fig. 6) revealed that the *CT* surfaces consistently showed a substrate-like profile dominated by zirconium and oxygen, with yttrium and hafnium as minor constituents. The relative proportions fluctuated slightly over time but without qualitative shifts. After *AL* abrasion, the composition remained dominated by zirconium and oxygen, with aluminum present only at low levels and silicon in small amounts; the pattern was stable, with at most modest mid-term changes. In the *SI* condition, a persistent silica signal was present at all storage times alongside zirconium and oxygen, with aluminum remaining low and yttrium/hafnium within narrow ranges; the silica-enriched profile was maintained throughout storage. Following etching, fluorine and nitrogen were detectable and tended to decline with storage, approaching minimal or occasional trace levels by the later time points. In *AL-ET*, low-level aluminum from abrasion persisted, and fluorine and nitrogen were initially present but decreased over time. The *LS* group exhibited a profile with zirconium and oxygen predominant, and yttrium, aluminum, and hafnium as minor constituents. This composition remained qualitatively stable from Baseline to 1 year.

## Discussion

This study showed that surface-modification protocols and storage time jointly modulate wettability, surface free energy (SFE), work of adhesion (WoA), and near-surface chemistry of zirconia, thereby rejecting both null hypotheses. *Tribochemical silica coating (SiO₂*,* 30 μm)* consistently delivered the most hydrophilic profile, lower water contact angles, higher WoA, and higher total SFE with robust polar and dispersive contributions. Chemical etching, alone or after alumina abrasion, produced higher contact angles and lower polar SFE. *LS* and *CT* remained intermediate, and *AL* (110 μm) generally approached *SI* but showed a short-term inconsistency at one month. Effect-size analysis showed that surface conditioning was the dominant factor in wettability and SFE outcomes, accounting for substantially more variance than storage time, indicating that surface chemistry, rather than aging effects, governed wetting behavior. This distinction reflects the different physical nature of the investigated parameters: surface roughness is a directly measured topographical property, whereas surface free energy and work of adhesion are derived energetic parameters that depend on paired contact angle measurements.

The superiority of the SI protocol reflects a chemistry-dominated mechanism, inherent in tribochemical silica application with smaller particles [11, 13]. The process deposits a silica-rich layer with a higher density of surface silanol (–Si–OH) groups, increasing the polar component of SFE and lowering the contact angle, thereby enhancing aqueous and protein interactions at the interface [16, 19]. Concurrently, the 30 μm particles tend to produce a finer, more uniformly distributed microtexture (shallower, more frequent pits) that increases the real surface area without deep cratering, although effect-size analysis indicates that microroughness differences among non-laser groups contributed minimally to wetting behavior, thereby amplifying intrinsic hydrophilicity when surface chemistry is favorable [13, 20]. In contrast, 110 μm Al₂O₃ predominantly induces deeper cratering and localized plastic deformation with limited chemical activation (low embedded Al on EDS), which can raise roughness but does not increase the polar SFE to the same extent, explaining its generally intermediate wetting performance relative to SiO₂ [4, 13, 20].

Storage time produced treatment-specific trajectories consistent with time-dependent surface chemistry [11, 21]. *CT* specimens became progressively more hydrophilic, with increased total SFE, mainly polar, at 6 and 12 months, consistent with spontaneous hydroxylation “aging” in aqueous media that increases surface polarity [11, 21]. However, these changes that occur over time can also be affected by the unintentional buildup of airborne substances, such as hydrocarbons. These substances can gradually change how a surface behaves, including how it interacts with liquids. Nevertheless, effect-size estimates indicate that storage-time effects were secondary in magnitude compared with surface-conditioning effects. *The SI group* showed early hydrophilization at 1 month relative to baseline and maintained superior performance through 12 months, indicating durable silica-mediated surface activity despite modest fluctuations [4, 11, 21]. In contrast, *the ET group* began in a more hydrophobic/low-polar state at baseline and improved thereafter, mirroring elemental findings of initially detectable fluorine and nitrogen that declined with storage. Attenuation of etchant-related residues likely underpins the delayed wetting improvement [4, 11, 21]. The *AL-ET* condition, on the other hand, exhibited a similar early chemistry-driven shift followed by a stable plateau, whereas laser-treated specimens remained notably stable throughout the year, suggesting parameters that yielded a chemically stable surface with balanced polar/dispersive contributions [13, 20].

Elemental analysis helped contextualize these findings in terms of surface chemistry [4]. *Specimens in the SI gro*up retained a persistent silica signal at all time points, consistent with sustained hydrophilicity and elevated polar SFE [11, 21]. *The AL group showed* only low-level aluminum, with spectra dominated by Zr and O (minor Y/Hf), which explains why its wetting improvement was less uniformly maintained than that o*f the SI group* and suggests that its apparent convergence in total SFE was driven primarily by dispersive rather than polar contributions [13, 20]. *ET* groups initially exhibited fluorine and nitrogen, consistent with etchant byproducts, that waned over time, paralleling the shift toward lower contact angles and higher SFE at later time points [4, 11, 21]. *CT and LS* specimens showed substrate-like spectra with qualitative stability, matching their intermediate and temporally robust wetting profiles [13, 20]. It should be emphasized that EDS is a semi-quantitative, near-surface technique with limited sensitivity to light elements and a sampling depth on the order of micrometers; small fluctuations in minor constituents (e.g., ± 0.5% in Hf) therefore fall within expected analytical variability and warrant cautious interpretation of absolute values, while relative trends across treatments and time remain informative.

The observation that the CT group exhibited slightly higher roughness values than some conditioned groups (ET, SI, AL, and AL-ET) can be attributed to the fact that polishing-induced surface features of untreated zirconia may persist as broad, low-frequency topographical variations. In contrast, air abrasion is characterized by the impact of aluminum oxide particles that promote erosion of the zirconia substrate [22, 23], modifying the surface topography by selectively eroding high surface peaks remaining after polishing procedures [24]. This process reduces deep grooves and leads to a more uniform and standardized distribution of surface defects, as previously described in the literature [24]. Consequently, certain conditioning protocols may partially smooth the surface at the microscale while simultaneously altering its chemical composition. This finding underscores that higher roughness values do not necessarily translate into superior wettability or biological performance in the absence of favorable surface chemistry*.*

In the broader literature, these findings reinforce that not only microtopography but also surface chemistry and polarity govern the earliest biological interactions at the implant interface [4, 12, 13]. The present results align with previous reports demonstrating that tribochemical silica coating enhances zirconia wettability and polar SFE more effectively than alumina abrasion or etching alone, while also extending these observations by showing the temporal stability of these effects over one year. The dominance of polar SFE effects observed here suggests that early protein adsorption kinetics, rather than purely mechanical interlocking, are likely the principal mediators of the initial biological response. Surfaces that combine high total SFE with substantial polar components, exemplified here by 30 μm tribochemical silica, are better aligned with the hydrophilic, protein-compatible state associated with a faster transition from primary mechanical stability to secondary biological stability [5, 6, 8, 10, 21, 22]. Airborne-particle abrasion with 110 μm alumina can move zirconia in the same direction but appears more susceptible to short-term reconditioning, while certain etching chemistries may initially depress polarity/hydrophilicity due to transient residues unless additional rinsing/neutralization or pre-aging is provided [4, 11, 13, 21]. Laser texturing, when tuned to avoid adverse chemical alterations, can provide a mid-range yet temporally robust energetic state with predictable behavior [13, 20].

Clinically, steering zirconia toward higher polar SFE appears advantageous for early blood/protein interactions and osteogenic responses at the bone-implant interface [8, 21, 22, 25]. In the current context, 30 μm tribochemical silica yielded the most consistently hydrophilic/high-SFE surface over one year, whereas 110 μm alumina abrasion was generally beneficial but showed a short-term dip that might be mitigated by immediate post-treatment cleaning or adjusted timing [11, 13, 21]. Etched surfaces may benefit from more aggressive rinsing/neutralization or pre-aging to minimize residue-related hydrophobicity, and laser protocols can deliver temporally stable energetics if parameters are optimized [13, 20]. Limitations include the in vitro nature of wettability/SFE assessments using two probe liquids and the OWRK model, the semi-quantitative and depth-limited character of EDS, and the absence of direct protein/cell or in vivo outcomes. Future work should integrate high-resolution surface chemistry (e.g., XPS/ToF-SIMS) with biological readouts to link specific chemical states (e.g., silanol density or residual fluoride species) to functional osseointegration endpoints.

## Conclusions

From this study, the following could be concluded:

1. Zirconia surface characteristics were influenced by both the applied surface-modification protocol and storage time.
2. Among the evaluated treatments, tribochemical silica coating produced the most favorable wettability and surface free energy profile over time.
3. Alumina airborne-particle abrasion also improved surface properties compared with untreated zirconia, although its effects were less pronounced.
4. Etching protocols showed lower initial wettability with gradual improvement during storage, whereas laser texturing resulted in intermediate yet temporally stable surface characteristics.
5. Overall, the surface-modification strategy played a more relevant role than storage conditions in determining zirconia surface wettability and energetic behavior, which may be clinically relevant for early biological interactions at the tissue-implant interface.

## Data Availability

Datasets used and/or analyzed during this study are available from the authors upon reasonable request.
